# Antitumor Efficacy and Immunomodulation of H‑Ferritin Nanocaged Doxorubicin for Triple Negative Breast Cancer

**DOI:** 10.1021/acsanm.5c03120

**Published:** 2025-11-03

**Authors:** Marta Truffi, Leopoldo Sitia, Serena Mazzucchelli, Marta Sevieri, Arianna Bonizzi, Francesco Mainini, Raffaele Allevi, Simone Canesi, Camilla Recordati, Angelica Stranieri, Saverio Paltrinieri, Carlo Francesco Morasso, Francesca Baldelli Bombelli, Fabio Corsi

**Affiliations:** † 9292 Istituti Clinici Scientifici Maugeri IRCCS, via Maugeri 4, 27100 Pavia, Italy; ‡ Dipartimento di Scienze Biomediche e Cliniche, Università di Milano, via G.B. Grassi 74, 20157 Milano, Italy; § Mouse and Animal Pathology Laboratory, Fondazione Unimi, viale Ortles 22/4, 20139 Milano, Italy; ∥ Dipartimento di Medicina Veterinaria e Scienze Animali, 9304 Università di Milano, via dell’Università 6, 26900 Lodi, Italy; ⊥ SupraBioNanoLab, Department of Chemistry, Materials, and Chemical Engineering “Giulio Natta”, 18981 Politecnico di Milano, Milano, 20131, Italy

**Keywords:** H-ferritin nanoparticles, doxorubicin, triple negative breast cancer, patient-derived xenograft, drug delivery, toxicity

## Abstract

Triple-negative breast cancer (TNBC) remains a major therapeutic challenge due to its aggressiveness and lack of targeted treatment options. Ferritin-encapsulated doxorubicin (HFn-Dox) is a nanocarrier-based formulation with documented tumor targeting activity and antitumor potential. In this study, we advance its clinical relevance by evaluating the HFn-Dox efficacy and toxicity profile at therapeutic dosing in patient-derived xenograft (PDX) and syngeneic TNBC models. HFn-Dox significantly outperformed free Dox by suppressing tumor growth and reducing metastatic spreading in both the models. When compared with free Dox and the clinically approved pegylated liposomal doxorubicin (Lipo-Dox), HFn-Dox also displayed a more favorable cardiotoxicity profile, which allowed dose intensification without compromising safety. Additionally, HFn-Dox modulated the tumor immune microenvironment in immunocompetent mice by enhancing intratumoral infiltration of Tlymphocytes and M1 macrophage polarization. In vitro, HFn-Dox preserved the T cell viability and prevented exhaustion. It also promoted the activation of macrophages and dendritic cells, contrasting with the immunosuppressive effects of free Dox. Altogether, our results demonstrate that HFn-Dox can increase the therapeutic index of doxorubicin by combining improved tumor delivery, reduced off-target toxicity, and immune system preservation. These features support the translational potential of HFn-Dox as a safer and more effective nanochemotherapy for TNBC.

## Graphical abstract

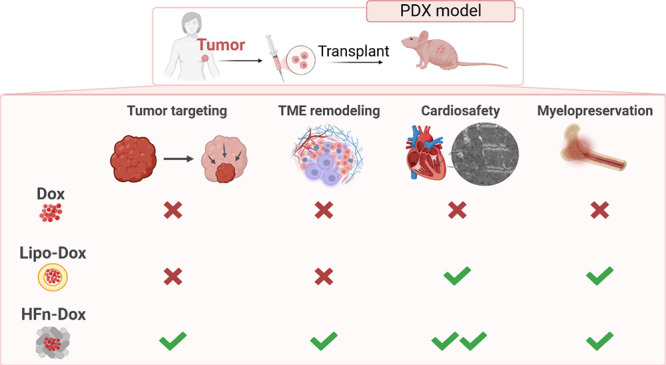

## 1 Introduction

Triple-negative breast cancer (TNBC) is characterized by the absence of estrogen receptor, progesterone receptor, and human epidermal growth factor receptor 2 overexpression, making it refractory to conventional targeted therapies.^1,2^ Doxorubicin (Dox)-based chemotherapy remains the mainstay of treatment for TNBC in neoadjuvant, adjuvant, and metastatic settings. However, Dox has a significant usage limitation related to the patient’s body surface area.^3,4^ A dosage limit is established to prevent the risk of irreversible cardiac damage and myelosuppression, necessitating careful monitoring of the total administered dose throughout the treatment.^5–7^ Moreover, high-dose chemotherapy often causes severe immunosuppression due to cytotoxic effects on healthy proliferating cells, including those in the immune system.^8,9^ In this regard, Dox is exemplary of a class of antineoplastic drugs that would benefit from the design and advancement of drug delivery systems^10,11^ that enhance selective tumor targeting and reduce off target accumulation of the drug.

Dox formulation exploiting H-Ferritin (HFn) nanocages has been developed and optimized over the years by our group showing to be an optimal candidate for clinical translation in TNBC therapy.^12–15^ HFn are natural spheric nanoparticles with a uniform-size cavity that can accommodate different kinds of drugs.^16,17^ They are internalized in the cells upon specific binding with the Transferrin receptor 1 (TfR1), overexpressed in almost all types of human cancer, thus, ensuring natural homing to solid tumors. The strengths of HFn include biocompatibility, biodegradability, tumor targeting capability, and ability to encapsulate a variety of therapeutic payloads. We already demonstrated the antitumor potential of Dox-loaded HFn (HFn-Dox) in vitro^12,13^ and tested its efficacy in a nanometronomic setting in 4T1-bearing mice.^14^ HFn-Dox administered at repeated low doses arrested tumor progression, inhibited tumor angiogenesis, avoided chemoresistance, and overcame cardiotoxicity, thus suggesting HFn-Dox nanometronomic chemotherapy as a safer and more effective oncological treatment.^14,18^

In the present study, we aimed to test if HFn-Dox administered at a therapeutic standard dose could achieve antitumor efficacy while still maintaining a favorable toxicity profile. An in vivo study was conducted in a patient-derived xenograft (PDX) model derived from a primary chemotherapy naïve TNBC human sample, to make efficacy and tolerability data more reliable from a clinical translational perspective compared to existing literature. We also evaluated the immunomodulatory potential of HFn-Dox in a syngeneic TNBC model established in immunocompetent mice. The results obtained supported the enhanced therapeutic performance of HFn-Dox mainly due to reduced side effects on cardiac and immune cells further improving the efficacy/tolerability balance in a standard-dosed nanochemotherapy.

## 2 Materials and Methods

### 2.1 HFn-Dox Production

HFn was obtained as a recombinant protein in pET11a/HFn transformed ClearColi BL21 (DE3) cells (Lucigen, LGC Ltd., UK) following a protocol we recently optimized.^13,19^ First, protein production was induced with Isopropyl β-D-1-thiogalactopyranoside (IPTG, 0.5 mM) by overnight incubation (O/N). Then, cells were lysed and heat-treated (70 °C, 15 min), and HFn was purified by ion-exchange chromatography (Q-Sepharose resin, cat. no. 17051010, Cytiva) with an increasing stepwise gradient of NaCl in 20 Mm Tris-HCl pH 8.0 buffer. Endotoxins (LPS) were removed by incubating HFn with Triton X-114 (1% v/v in dH_2_O), as already described by our group.^13^ HFn was dosed by A280 absorbance reading and Bradford and BCA assays. LPS were quantified using the LAL kinetic turbidimetric assay following the manufacturer’s instructions (Charles River Microbial Solutions Ltd., Dublin, Ireland). Dox was loaded into HFn by exploiting the pH disassembly reassembly strategy, as already described by our group.^20^ The nanocages were disassembled by adjusting the pH to 2 for 15 min. Then, Dox (200 μM, Teva Pharmaceuticals, USA) was added, and the pH was adjusted to 7.5. After 2 h of incubation at 180 rpm at room temperature (RT), the nanodrugs (HFn-Dox) were concentrated up to the desired Dox concentration using 100 kDa Amicon membranes (Merck Millipore) and the unloaded drug was removed using 7 k MWCO Zeba Spin Desalting columns (Thermo Fisher). Final Dox content was determined by spectrofluorimetry (FP-800, Jasco) after Dox extraction in a 1:1 isopropanol chloroform solution, using a standard curve obtained with free Dox at different concentrations as a reference.^14^ Moreover, the number of Dox molecules/nanocage, the encapsulation efficiency (encapsulated Dox: used Dox mass ratio (%)) and the loading capacity (Dox:HFn mass ratio (%) in the final preparation) have been calculated. The different batches of HFn-Dox were aliquoted and stored at – 20 °C for up to 4 weeks. Before every experiment, a reference aliquot was thawed, and its stability was characterized as described below, as an internal quality control.

### 2.2 HFn-Dox Characterization

HFn was characterized by sodium dodecyl sulfate-polyacrylamide gel electrophoresis (SDS-PAGE, (12% gel with a Coomassie brilliant blue protein stainer) to evaluate protein purity after LPS removal. Both HFn and HFn-Dox nanodrugs morphology was studied by transmission electron microscopy (TEM, Tecnai Spirit, FEI, Hillsboro, OR, USA) by negatively staining proteins with uranil-acetate 1% for 30 s at RT at 80 k–135 k × and 220 k–300 k × magnification. Finally, Dynamic Light Scattering (DLS) was used to evaluate the HFn and HFn-Dox hydrodynamic size. Measurements (three measurements/sample) were performed at 25 °C with a scattering angle set at 90° using an ALV/CGS-3 Platform-based Goniometer System equipped with an ALV-7004 correlator, an ALV/CGS-3 goniometer, and a diode-pumped Coherent Innova Nd:YAG laser (λ = 532 nm). Stability of HFn-Dox was evaluated by thawing aliquots of the frozen nanodrugs at predetermined time points (1, 7, 14, 21, and 28 days after preparation). After 5′ of centrifuge (1 × 10^4^ *g*, 4 °C), HFn and Dox concentrations were determined by absorbance reading (Protein and Labels function, NanoDrop 2000c Spectrophotometer, ThermoFisher Scientific) and by spectrofluorimetry (as described above) respectively.

### 2.3 PDX Study

HBCx-17 PDX study was carried out at XenTech (France) in accordance with French regulatory legislation concerning the protection of laboratory animals. Athymic Nude-Foxn1^nu^ female mice aged 6-to-9 weeks (*ENVIGO*, Gannat, France) were allocated to acclimate in a specific pathogen-free animal facility (Center for Exploration and Experimental Functional Research (CERFE, Evry, France)) with access to food and water *ad libitum* for at least 6 days prior to tumor implantation. Mice were grouped-housed (maximum of 6 animals) in Polysulfone plastic individually ventilated cages (IVC) bedded with sterilized and dust-free bedding cobs. Primary chemotherapy-naïve TNBC samples, namely the HBCx-17 PDX model, were implanted subcutaneously. Animals were included in the study 28 days postimplantation (day 0), and treatments were initiated on the same day. Only mice with an established growing tumor of size between 63 and 221 mm^3^ were allocated to the treatment groups (n = 9). Groups were standardized to mean and median tumor volume of 121 and 108 mm^3^ respectively. HFn-Dox (2.5 and 5 mg/kg), free Dox (2.5 mg/kg), or pegylated liposomal formulation of Dox called Lipo-Dox (Caelyx, 2.5 mg/kg) were dosed by slow i.v. injection 2qwk × 3. Injectable sterile saline solution (NaCl 0.9%) was administered to a group of mice and used as a negative control. During the experimental period, all mice were observed clinically for physical appearance, behavior, and clinical changes; tumors were measured with caliper and mice were weighed twice a week. At study end (day 21), 400 to 600 μL of whole blood were collected by intracardiac puncture under xylazine-ketamine anesthesia. Blood was transferred into K3EDTA-coated tubes and then centrifuged at 5000 rpm (centrifuge Eppendorf 5425R rotor FA-24 × 2) at room temperature for 5 min. Plasma was collected in polypropylene tubes and frozen at −80 °C for subsequent clinical chemistry analysis. Tumors were excised from treated animals, weighted *ex vivo* and fixed in 10% formalin. Hearts were cut longitudinally and fixed in either 2.5% glutaraldehyde solution (half) or 10% formalin (half) for TEM imaging and histology, respectively. All other off target organs (spleen, femurs, liver, kidneys, gut, lungs, brain; n = 4/group) were collected for histological evaluation.

### 2.4 In Vivo Treatment of 4T1 Mouse Model

Female BALB/c mice aged 8 weeks old (Charles River Laboratories, Calco, Italy) were maintained in the animal facility, fed ad libitum, and allowed to acclimate for 1 week before tumor implantation. In vivo study complied with relevant ethical regulations for animal testing and research and received ethical approval by the Italian Ministry of Health (aut. N. 110/2018-PR). In any case, the maximal tumor size permitted by the ethics committee, i.e., 1.5 cm average diameter, was not exceeded. Mice were grouped-housed in individually ventilated cages (IVC), and subjected to abdominal trichotomy 2 days before the injection of tumor cells upon gas anesthesia with 2.5% isoflurane. 4T1-Luc2 cells (1 × 10^5^ cells) were suspended in cold serum-free RPMI 1640 medium and orthotopically injected into the mammary fat pad. About 4 days after implant, when a small primary nodule was visible, tumor size was measured with caliper and mice were weighted to collect baseline parameters. Then, mice were divided into four groups with homogeneous mean tumor volume (n = 10) and treated with saline solution used as placebo, HFn-Dox (5 mg/kg), free Dox (5 mg/kg), or Lipo-Dox (2.5 mg/kg) by intravenous (i.v.) injection into the tail vein. Treatment was repeated four times (i.e., day 5, 7, 11, 15 after tumor implant), and animals were sacrificed at day 18 to assess antitumor activity and toxicity of treatments. The day before each injection, tumor volume was evaluated by measuring the tumor diameters with a caliper and mice were weighted to monitor treatment efficacy and animal wellness. Tumor volume was determined using the following equation: (length × width^2^)/2. Moreover, bioluminescence images (BLI) of 4T1 tumors were acquired 10 min after intraperitoneal injection of luciferin (150 μg/kg, PerkinElmer) by the IVIS Lumina II system. At the end of treatment, blood samples were drawn from the retro-orbital plexus and collected in EDTA-coated tubes for hematological analysis by Idexx Procyte analyzers (Laboratorio Analisi Murine, IRCCS San Raffaele, Milano, Italy). Mice were euthanized by cervical dislocation upon gas anesthesia. Tumor, spleen, and femurs were dissected and either fixed in 10% neutral buffered formalin (NBF) (n = 4/group) or soaked with cold PBS for cell dissociation (n = 6). Hearts were resected from 8 mice/group and fixed in NBF (n = 4) or 2.5% glutaraldehyde (n = 4) for histology and transmission electron microscopy (TEM), respectively. Liver, kidneys, lungs, and gut were resected from 4 mice/group and fixed in NBF for histology.

### 2.5 Histology Assessment

Tissue samples obtained from treated mice were fixed in NBF for 24–48 h and routinely processed for paraffin embedding. Sections of 4 μm thickness were cut, stained with hematoxylin-eosin, and examined blindly.

### 2.6 Immunohistochemistry

For immunohistochemistry, tumor sections were deparaffinized and underwent heat-induced epitope retrieval at pH 9, for 40 min at 95 °C (Dewax and HIER Buffer H, TA-999-DHBH, Thermo Scientific, UK). Endogenous peroxidase activity was blocked by incubating sections in 3% H_2_O_2_ for 10 min. Slides were rinsed and treated with PBS containing 10% normal rabbit serum for 30 min to reduce nonspecific background staining and then incubated for 1 h at room temperature with anti-MHC I (Abcam, ab52922, 1:600) and γH2AX (Ser139) (Cell Signaling, clone 20E3, #9718,1:2000). Sections were incubated for 30 min with appropriate biotinylated secondary antibody (Vector Laboratories, Burlingame, CA, USA, 1:200), and then labeled by the avidin–biotin-peroxidase (ABC) procedure with a commercial immunoperoxidase kit (VECTASTAIN Elite ABC HRP Kit Standard, PK-6100, Vector Laboratories, Burlingame, CA, USA, 1:150). The immunoreaction was visualized with 3,3′- diaminobenzidine substrate (Peroxidase DAB Substrate Kit, VC-SK-4100-KI01, Vector Laboratories, Burlingame, CA, USA) for 5 min, and sections were counterstained with Mayer’s hematoxylin. Every immunohistochemical run included a suitable positive control to control the staining procedure. Slides were digitalized through a NanoZoomer S60 Digital slide scanner (Hamamatsu Photonics K.K., Hamamatsu City, Japan). Image analysis was performed by using QuPath v0.3.2 image analysis software. In each sample, the entire section was manually annotated to obtain the tumor area (necrotic areas were discarded). To evaluate the positivity, the number of γH2AX+ cells was assessed in the annotated regions using the ‘Cell detection’ algorithm. The number of positive cells and tumor area was then calculated.

### 2.7 Clinical Chemistry Analysis

Clinical chemistry analysis was performed on plasma samples using an automated spectrophotometer (BT3500, Biotecnica Instruments SPA, Roma, Italy) and measuring the following analytes using reagents provided by Futurlab S.r.l. (Limena, PD, Italy): albumin (bromochresol green method), creatinine (Jaffè method), alanine aminotransferase (ALT, Kinetic IFCC method), and glutamate dehydrogenase (GLDH, DGKC method).

### 2.8 Flow Cytometry of Dissociated Tumors

4T1 tumors surgically removed from treated mice (*n* = 6/group) were dissociated into single cell suspension using tumor dissociation kit (#130-096-730, Miltenyi Biotec) and gentleMACS tissue dissociator (#130-093-235, Miltenyi Biotec) following the manufacturer’s instructions. Isolated cells (5 × 10^5^/tube) were washed with cold PBS and stained with Live/Dead (L34976; Thermo Scientific). To identify specific cell subpopulations, appropriate mixtures of fluorescence-conjugated antibodies were prepared in FACS buffer (PBS, 2% Fetal Bovine Serum) and incubated with the cell suspensions: Ab panel 1) CD45-ef506, CD11b-AF488, CD11c-PE-Cy5, MHCII-sb436, F4/80-AF700, Ly6C-PE-Cy7, LY6G-APC; Ab panel 2) CD45-ef506, CD3-PE-Cy7, CD4-APC, CD8a-AF488, CD19-sb436; Ab panel 3) CD3-PE-Cy7, CD4-APC, CD8a-AF488, CD69-PE-Cy5, CD44-AF700, CD62l-APC, PD1-sb436, CTA5-APC; Ab panel 4) CD45-ef506, CD11c-PE-Cy5, F4/80-AF700, CD86-sb436, CD80-AF488, ARG1-PE-Cy7, iNOS-PE, CD206-APC. For panel 4, Ab incubation was performed in permeabilization buffer (00–8333–56, Thermo Fisher Scientific). Data were collected on a CytoFLEX cytometer equipped with blue yellow and violet lasers (Beckman Coulter) by gating on viable singlets. Unstained cells were used to set the region of positivity. The gating strategy used for the analysis is reported as Supporting Information (see Supporting Figure S1). All antibodies were purchased from Thermo Fisher Scientific.

### 2.9 Analysis of Splenocytes and Bone Marrow Cells

Spleens from 4T1-bearing mice (n = 6/group) were collected, freshly smashed on a 70 μm CellStrainer filter, and treated with the ACK lysis buffer (Euroclone) to lyse red blood cells. Isolated splenocytes were stained with Live/Dead and antibody panels 1 and 3 for flow cytometry analysis, as described above. Fresh bone marrow cells were collected from the femur: the proximal and distal epiphysis were removed, and the medullary cavity was flushed with saline solution until bone marrow was extracted. Then, bone marrow was freshly smashed and flushed through a CellStrainer filter. The collected cells were stained with Live/Dead and with antibody panel 1 and acquired with CytoFLEX, as described above.

### 2.10 Toxicity Analysis in Heart Tissue

Side effects of treatments were evaluated in the heart by TEM. Heart tissues excised from 4T1 and PDX mouse models were fixed in 2.5% glutaraldehyde in 0.1 M phosphate buffer, then fixed in 1.5% osmium tetroxide for 2 h, dehydrated with EtOH scale, and embedded in epoxy resin (PolyBed 812 Polysciences Inc.). Ultrathin sections were cut, stained with uranyl acetate and examined by TEM (Tecnai Spirit, FEI) as previously described.^21^ Mitochondria morphometric measurements were performed using ImageJ software on at least 10 images/group (4,200× magnification) and measuring at least 100 mitochondria/group. Details about image analysis can be found in.^18^

### 2.11 In Vitro Immunostimulation Assay

Mouse splenic T cells and bone marrow-derived monocytes were collected from wild type BALB/c female mice (authorization number 110/2018), as described above, cultured in vitro, and treated to assess the direct effect of different Dox formulations. Bone marrow-derived monocytes were cultured for 5 days with the addition of M-CSF or GM-CSF in the culture medium to induce differentiation into dendritic cells (DC) or macrophages (M), respectively. Then, cells were treated with the compounds (HFn-Dox, free Dox and Lipo-Dox) at a final concentration of 0.1 μM Dox equivalent for 24 h at 37 °C. As a positive control, LPS+IFNg was used to polarize differentiated cells toward the M1 phenotype. At the end of treatment, the cells were collected in FACS tubes, washed, and analyzed by flow cytometry to measure viability and frequency distribution of different cell populations. In the case of splenic T cells and bone marrow-derived dendritic cells, staining was performed with Live/Dead and with antibody panel 4. After cells were washed three times with PBS, tubes were acquired with CytoFLEX, as described above.

### 2.12 Statistical Analysis

Data were reported as mean ± standard deviation (SD) or standard error of the mean (SE), as indicated. For in vivo efficacy studies, statistical analysis was done for each measurement by Student *t* test or Mann–Whitney non parametric comparison test in case of normal and non-normal distribution of the data, respectively. Each treated group was compared to the control group and to the other treatments. When multiple comparisons were assessed the ANOVA or the Kruskal–Wallis test was applied. Graphs were generated using GraphPad Prism 6 (San Diego, CA, USA). Statistical significance was set at p-value <0.05.

## 3 Results

### 3.1 Antitumor Efficacy of HFn-Dox in PDX Model

Nonpyrogenic HFn-Dox nanodrugs were produced using protocols previously described,^12,19^ to obtain highly reproducible batches of HFn-Dox clear suspension with a final drug concentration of 0.51 mg/mL (±0.18), an encapsulation efficiency of 12.41% and a loading capacity of 3.43% (Figure 1A and Table 1), in line with the literature.^13,18,22^

**Table 1:** HFn-Dox Characterization (*n* = 5)

Dox (mg/mL)	Dox molecules/nanocage	Loading capacity (%)	Encapsulation Efficiency (%)	EU/mg
0.513 ± 0.18	30.17 ± 7.11	3.43 ± 0.81	12.41 ± 3.77	0.41 ± 0.26

**Figure 1:**
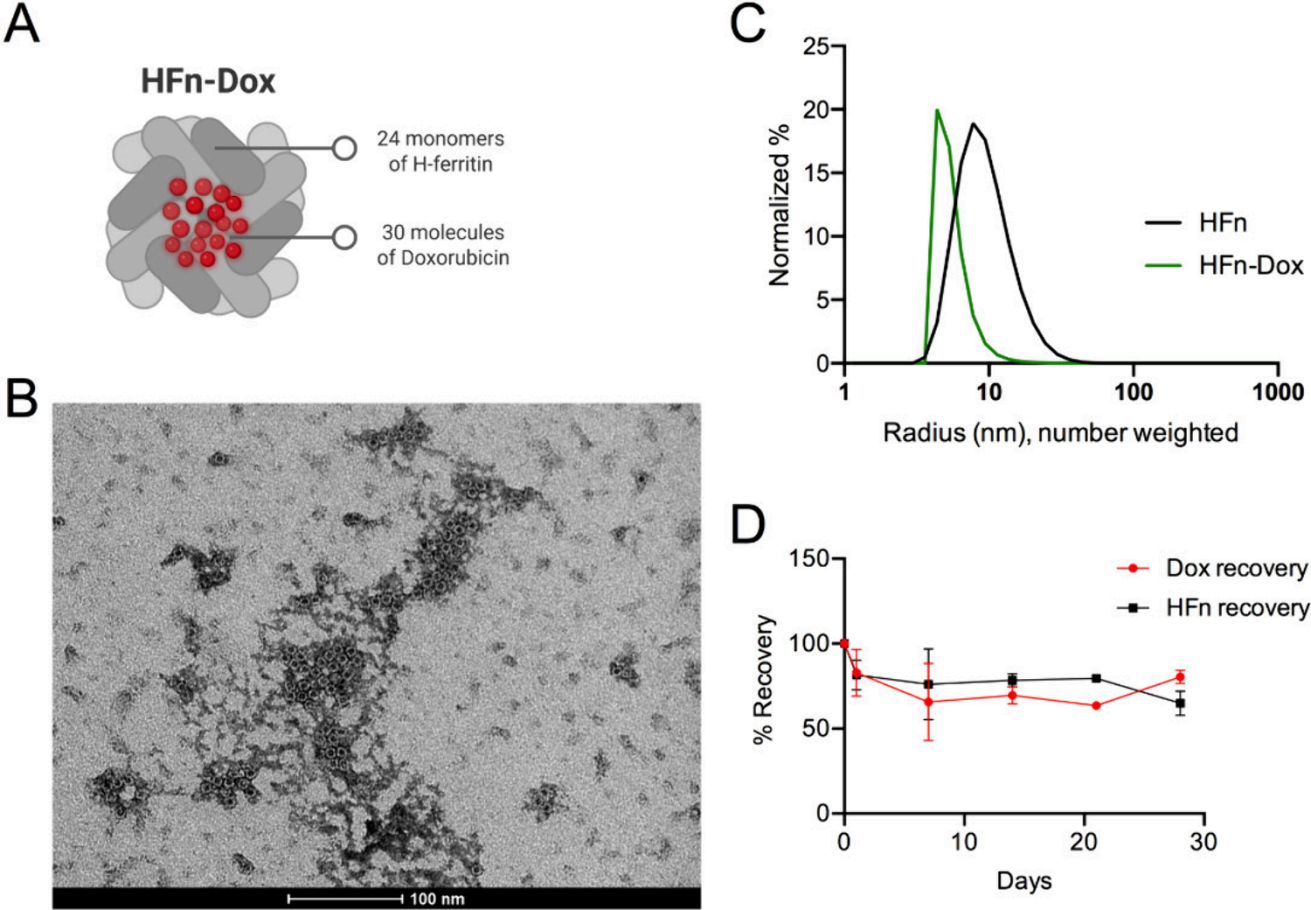
HFn-Dox characterization. A) Schematic representation of the nanoparticle structure, illustrating its main components and overall architecture; B) TEM representative image of HFn-Dox, where the characteristic nanocage structure is confirmed, scale bar = 100 nm; C) Normalized hydrodynamic radius distribution (number weighted) of HFn and HFn-Dox obtained by DLS analysis; D) Stability analysis of HFn-Dox in terms of protein and drug recovery after thawing the frozen nanodrug at different time points after preparation.

TEM confirmed the structural integrity of the particles (Figure 1B). DLS data revealed a predominant population with a size similar to that of empty HFn, approximately 10 nm; Figure 1C). Additionally, there was a second population consisting of slightly aggregated particles, with an average size of around 100 nm only visible in the intensity-weighted size distribution (Supporting Figure S2). The LAL test certified a concentration of endotoxins of 0.41 EU/mg. The nanodrug stability was checked by measuring the exact concentration of HFn and Dox in every single batch intended for the in vivo study (Figure 1D). Moreover, results obtained from the cell viability assessment (Supporting Figure S3) indicated a clear dose–response cytotoxic effect, both at 24 and 48 h, as already demonstrated by our group.^12,13^

The antitumor efficacy of HFn-Dox was assessed in the HBCx-17 PDX model, derived from a primary chemotherapy-naïve TNBC human sample. Mice were randomly divided into four experimental groups and treated with placebo, HFn-Dox, free Dox or a clinically approved pegylated liposomal Dox (Lipo-Dox), which is currently indicated for the treatment of advanced ovarian cancer and in some cases of metastatic breast cancer.^23–28^ The different compounds were administered at 2.5 mg/kg according to preliminary data about the PDX sensitivity to Dox and Lipo-Dox (XenTech confidential data). HFn-Dox was also tested at 5 mg/kg to evaluate nanodrug tolerability at a higher dose. The results indicated that HFn-Dox significantly reduced tumor burden to a similar extent as compared to free Dox and Lipo-Dox equally dosed (Figure 2A). Administration of HFn-Dox at 5 mg/kg further reduced the final tumor volume (*p* < 0.0001 vs placebo; *p* < 0.05 vs Dox) and induced tumor regression. At the end of treatment, tumor weight measured ex vivo was significantly decreased, especially in the group treated with HFn-Dox at 5 mg/kg (Figure 2B). Histological evaluation of the excised tumors revealed that HFn-Dox induced an increase in tumoral fibrovascular stroma (Figure 2C, *p* = 0.03 HFn-Dox 2.5 vs placebo; *p* < 0.0001 HFn-Dox 5 vs placebo) and inflammatory cell infiltration (Figure 2D, *p* < 0.0001 HFn-Dox 5 vs placebo), as well as a decrease in tumor cells’ mitotic activity (Figure 2E, *p* < 0.0001 HFn-Dox 5 vs placebo). By contrast, treatment with free Dox did not alter tumor stroma nor the number of mitosis. Results obtained with Lipo-Dox were less significant than those obtained with HFn-Dox (*p* < 0.01 vs placebo) for the same parameters. The metastatic spreading of the tumor cells was detected by histology in one mouse from the placebo group; thus, it was further investigated by staining murine organs with an antihuman MHCI antibody. The analysis revealed the presence of rare circulating tumor-derived cells in the lungs of all analyzed mice from the placebo group and in 25% of mice treated with free Dox. By contrast, no metastatic spreading of MHCI+ cells was detected in mice treated with HFn-Dox and Lipo-Dox (Supporting Figure S4). To better investigate the antitumor efficacy of the compounds, we performed γH2AX immunohistochemical analysis on fixed tumor sections as a marker of DNA double-strand breaks and DNA damage (Figure 2F,G). Overall positivity to γH2AX was not increased by the treatments, but there was a significantly different distribution of low, medium, and high positive cells for γH2AX expression among groups (Supporting Figure S5). Mice treated with HFn-Dox at 5 mg/kg had the most significant increase in high- and medium-positive γH2AX cells as compared to the other groups of treatment (Figure 2F), indicating superior efficacy of this treatment in inducing tumor cell damage.

**Figure 2:**
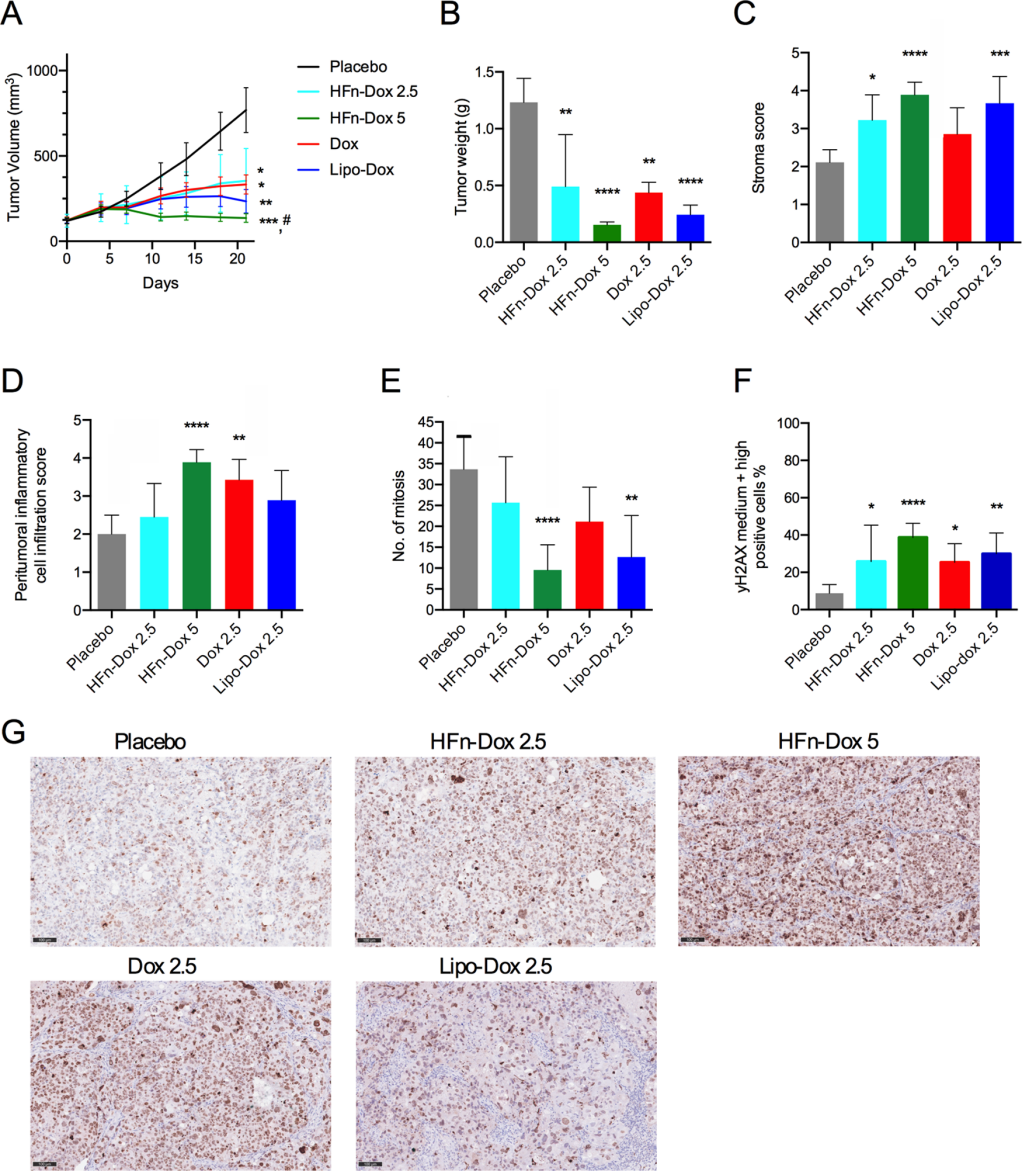
Efficacy of HFn-Dox treatment in the PDX model. A) Tumor volume was measured during treatment with placebo, HFn-Dox (2.5 and 5 mg/kg), free Dox (2.5 mg/kg), Lipo-Dox (2.5 mg/kg). *p < 0.05, **p < 0.01, ***p < 0.001, vs placebo; ^#^ *p* < 0.05 vs Dox by Mann–Whitney test B) Tumor weight ex vivo at the end of treatment. Plot reported means ± SE (*n* = 9). C-E) Histology score of tumor stroma, peritumoral inflammatory cell infiltration, and number of mitoses calculated in 3 fields at 400×. F-G) Immunohistochemistry of γH2AX in tumor sections. Scale bar = 100 μm. *p < 0.05, **p < 0.01, ***p < 0.001, ****p < 0.0001 vs placebo; ^#^ *p* < 0.05 vs Dox by one-way Anova.

During treatment, mice were constantly monitored for the appearance of clinical signs and adverse effects associated with drug administration. One mouse showed a distended abdomen after three injections of free Dox, and appeared emaciated, pale, and cold to the touch after the fourth injection, highlighting toxicity issues associated with free Dox. Such clinical observations imposed the ethical sacrifice of the animal at day 14 post-treatment; hence, for the Dox group, eight out of nine mice completed the chemotherapy cycle until day 21. No significant changes in body weight were found, although small changes in animal body weight (below 10%) were observed in all the treatment groups (Supporting Figure S6).

Heart tissues were analyzed for potential cardiotoxic effects associated with the treatments. The ultrastructural analysis uncovered severe mitochondria alterations in mice treated with Dox and Lipo-Dox (Figure 3A). Quantitative analysis of TEM images showed a marked increase in mitochondrial area in Dox and Lipo-Dox treatments (Figure 3B) as well as an increase in the percentage of damage (Figure 3C), which was measured as the area depleted of the mitochondrial cristae divided by the total mitochondrial area. By contrast, HFn-Dox treatment was not associated with mitochondrial alterations, either when dosed at 2.5 or 5 mg/kg, thus showing significant protection of the heart as compared with the other formulations.

**Figure 3:**
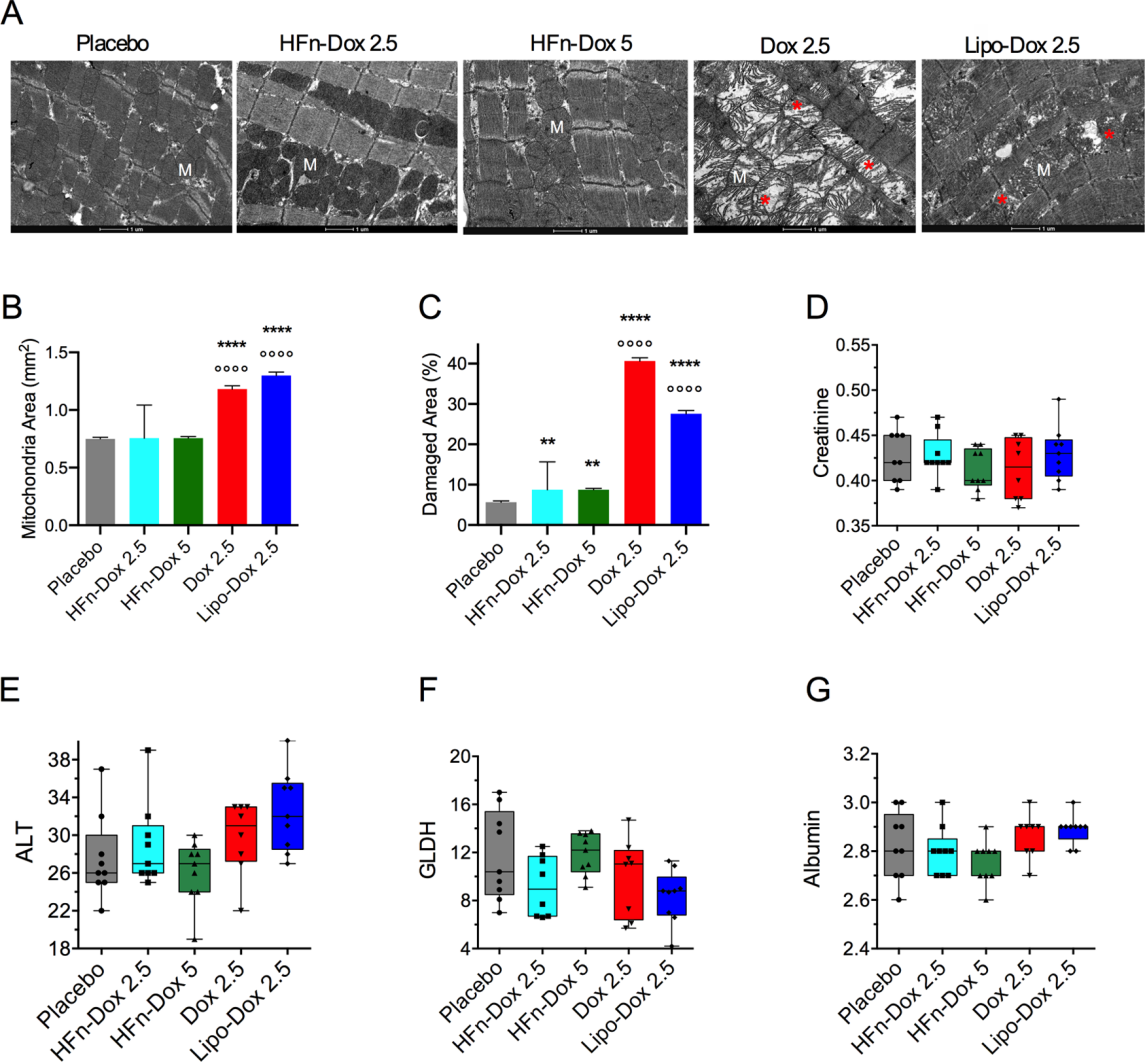
Toxicity analysis was performed in PDX. A) TEM representative images of the heart tissue at the end of treatment. M indicates mitochondria; asterisks (*) indicate damage to the mitochondrial cristae; scale bar = 1 μm; B) Cardiac mitochondria area and C) Percentage of damage in cardiac mitochondria were measured on TEM images from 4 mice/group and reported as mean of 10 fields of view (FOV) ± SE **p < 0.01, ****p < 0.0001 vs placebo; °°°°*p* < 0.0001 vs HFn-Dox treatment groups by one-way Anova. D-G) Plasmatic levels of creatinine (D), alanine aminotransferase (ALT, E), glutamate dehydrogenase (GLDH, F) and albumin (G) in the different treatment groups. Data are shown as box and whisker plots; no statistical significance of the treatment groups vs placebo was observed by Kruskal–Wallis test with multiple comparisons.

Additionally, the potential hepatic and renal toxicity was assessed, considering that these organs are preferential sites of nanoparticle accumulation.^14,22^ Histological analysis of liver and kidney sections showed no signs of structural damage, necrosis, or inflammation, supporting the absence of treatment-related effects (Supporting Figure S7). Furthermore, plasmatic measurements of creatinine (Figure 3D), alanine aminotransferase (ALT, Figure 3E), glutamate dehydrogenase (GLDH, Figure 3F), and albumin (Figure 3G) were performed. No alterations in these indices were observed following treatment with HFn-Dox at either 2.5 or 5 mg/kg. The hematochemical values, associated with the absence of histological lesions, tend to exclude phenomena of hepatotoxicity and nephrotoxicity.

### 3.2 Standard-Dosed HFn-Dox in 4T1 Syngeneic Model

After assessing the pharmacological efficacy of HFn-Dox in a PDX model, we proceed to a syngeneic immunocompetent mouse model to evaluate the nanodrug’s impact on the tumor immune microenvironment. This is crucial because we recently showed that HFn-Dox preserve viability and proliferative capacity of T lymphocytes in vitro.^20^ Here, we wanted to verify this effect in vivo, simulating a standard dose treatment in a clinically relevant setting. Immunocompetent BALB/c female mice bearing orthotopic 4T1-Luc2 TNBC were randomly divided into four experimental groups and treated with placebo, HFn-Dox, free Dox, or Lipo-Dox by repeated i.v. administrations. Drugs were dosed at 5 mg/kg except for Lipo-Dox which was administered at 2.5 mg/kg, according to the paradigm of minimum effective dose, as it is known that this formulation has prolonged circulation time and avoidance of the reticuloendothelial system.^29^

Tumor growth profiles reported in Figure 4A showed that HFn-Dox significantly reduced tumor volume as compared to placebo, but on the basis solely of tumor volume measurements, the therapeutic benefit of HFn-Dox did not surpass that of Lipo-Dox or free Dox. However, as tumor volume does not necessarily reflect the amount of viable tumor tissue, we also assessed tumor cell viability using bioluminescence imaging (BLI) at the end of treatment. Only tumors treated with HFn-Dox and Lipo-Dox, but not those treated with free Dox, showed a significant reduction in tumor’s BLI counts as compared to placebo (Figure 4B). These data revealed that HFn-Dox effectively reduced the population of viable tumor cells despite only modest changes in gross tumor volume. Moreover, BLI allowed distinguishing perfused, metabolically active regions from nonperfused necrotic areas, highlighting the functional impact of the treatment. Histological analysis further assessed these aspects of tumor severity and treatments efficacy. In particular, ulceration and necrosis are established indicators of tumor aggressiveness in the 4T1 model, and their reduction reflects a less aggressive tumor phenotype. HFn-Dox-treated tumors showed lower ulceration and necrosis compared to other groups, suggesting a less aggressive phenotype and better control of tumor growth (Table 2).

**Table 2:** Histological Assessment of Ulceration and Necrosis in Tumors from the 4T1 Model after Treatments

	Ulceration (%)	Necrosis score (median score)
Placebo	100	3
Dox	100	3.25
HFn-Dox	50	2
Lipo-Dox	75	4

**Figure 4:**
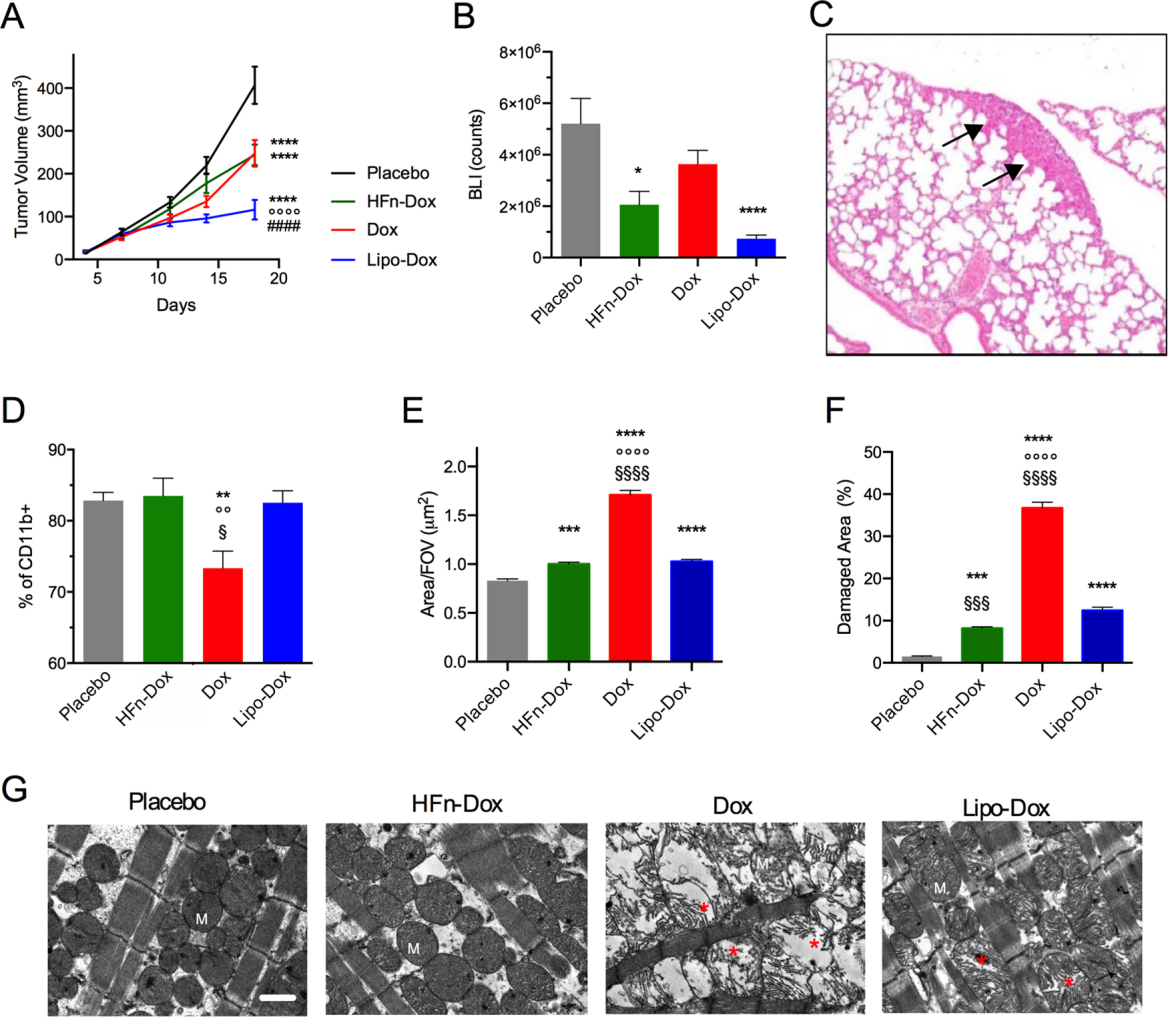
Efficacy of HFn-Dox treatment in a syngeneic TNBC model. A) Tumor progression in 4T1-tumor bearing BALB/c mice (*n* = 10/group) treated with placebo, HFn-Dox, Dox, Lipo-Dox. B) Bioluminescence signal (BLI) of 4T1-L cells 5 min after intraperitoneal injection of luciferin at the end of treatment (*n* = 10). C) Representative H&E of animal lungs showing metastases (arrows). D) Percentage of CD11b+ bone marrow cells extracted from treated mice was evaluated by flow cytometry. Data are means ± SE. Statistical significance *p = 0.02, **p < 0.01, ****p < 0.0001 vs placebo; °°*p* < 0.01, °°°°*p* < 0.0001 vs HFn-Dox; ^####^ *p* < 0.0001 vs Dox; ^§^ *p* < 0.05 vs Lipo-Dox by one-way Anova. E) Cardiac mitochondria area and F) Percentage of damage in cardiac mitochondria were measured on TEM images from 4 mice/group and reported as mean of 10 fields of view (FOV) ± SE; ***p < 0.001, ****p < 0.0001 vs placebo; °°°°*p* < 0.0001 vs HFn-Dox; ^§§§^ *p* < 0.001, ^§§§§^ *p* < 0.0001 vs Lipo-Dox by one-way Anova. G) TEM representative images of the heart tissue at the end of treatment, scale bar = 1 μm.

Tumor weight measured ex vivo confirmed reduction of the tumor mass upon treatment with all of the tested compounds (Supporting Figure S8). Tumor metastases of variable size and number were found in the lungs of the placebo- and Dox-treated groups (Figure 4C and Table 3). By contrast, no metastases were detected in HFn-Dox and Lipo-Dox groups, indicating better control of the metastatic spreading by nanoformulated vs free Dox.

**Table 3:** Lung Metastases Calculated by a Blind Pathologist in Different Treatment Groups (Mean)

Lung metastases score (mean n./mouse)			
	*d* < 0.5 mm	0.5 mm < *d* < 2 mm	Tot
Placebo	1.25	0.75	2
Dox	0.25	0	0.25
HFn-Dox	0	0	0
Lipo-Dox	0	0	0

When looking at mice wellness, we observed a slight but significant body weight loss in Dox-treated mice and reduced hemoglobin concentration in free Dox and Lipo-Dox groups as compared to the placebo. White blood cell count in peripheral blood was comparable in free Dox, HFn-Dox and Lipo-Dox groups, while reduced as compared to placebo (Supporting Figure S9), further confirming the overall efficacy of the three compounds.^30^ Treatment with free Dox also induced a significant reduction in the frequency of CD11b+ monocyte precursors from the bone marrow, while HFn-Dox and Lipo-Dox did not show a detrimental effect on bone marrow cells (Figure 4D). Moreover, severe alterations in the cardiac mitochondria ultrastructure were detected by TEM imaging in free Dox-treated mice (Figure 4G). Alteration of cardiac mitochondria was quantitatively measured as an increase in mitochondria area (Figure 4E) and in percentage of mitochondria damage (Figure 4F), showing detrimental effects in Dox-treated hearts. By contrast, HFn-Dox and Lipo-Dox formulations were associated with reduced cardiotoxicity. In particular, HFn-Dox significantly limited the percentage of mitochondrial damage as compared to both free Dox and Lipo-Dox, thus showing enhanced cardiosafety (Figure 4F).

Then, we explored whether HFn-Dox altered the key components of the tumor immune microenvironment by phenotyping the dissociated tumors at the end of therapy. Overall frequency of intratumor CD3+ lymphocytes was not modified by the treatments, although a trend toward an increase was observed in HFn-Dox tumors (Figure 5A). The percentages of CD4+ effectors (Figure 5B) and CD8+ cytotoxic lymphocyte subpopulations (Figure 5C) were significantly increased upon treatment with HFn-Dox, while they remained almost unaltered upon treatment with free Dox and Lipo-Dox. An overall increase of macrophages was also observed in tumors treated with HFn-Dox and Lipo-Dox as compared to free Dox (Figure 5D). Of note, we analyzed the two main subsets of activated macrophages, i.e., M1 and M2 subtypes, whose diversification results from a dynamic process known as macrophage polarization in response to microenvironmental signals. We found that HFn-Dox induced a significant increase in M1/M2 ratio as compared to both placebo and free Dox groups (Figure 5E). Prevalence of inflammatory M1 macrophages over M2-like immunosuppressive subtype suggests enhanced antitumor immune response in the HFn-Dox group. Analysis of splenic T lymphocytes also showed increased frequency of CD3+ and CD8+ cells especially in HFn-Dox treated group (Supporting Figure S10).

**Figure 5:**
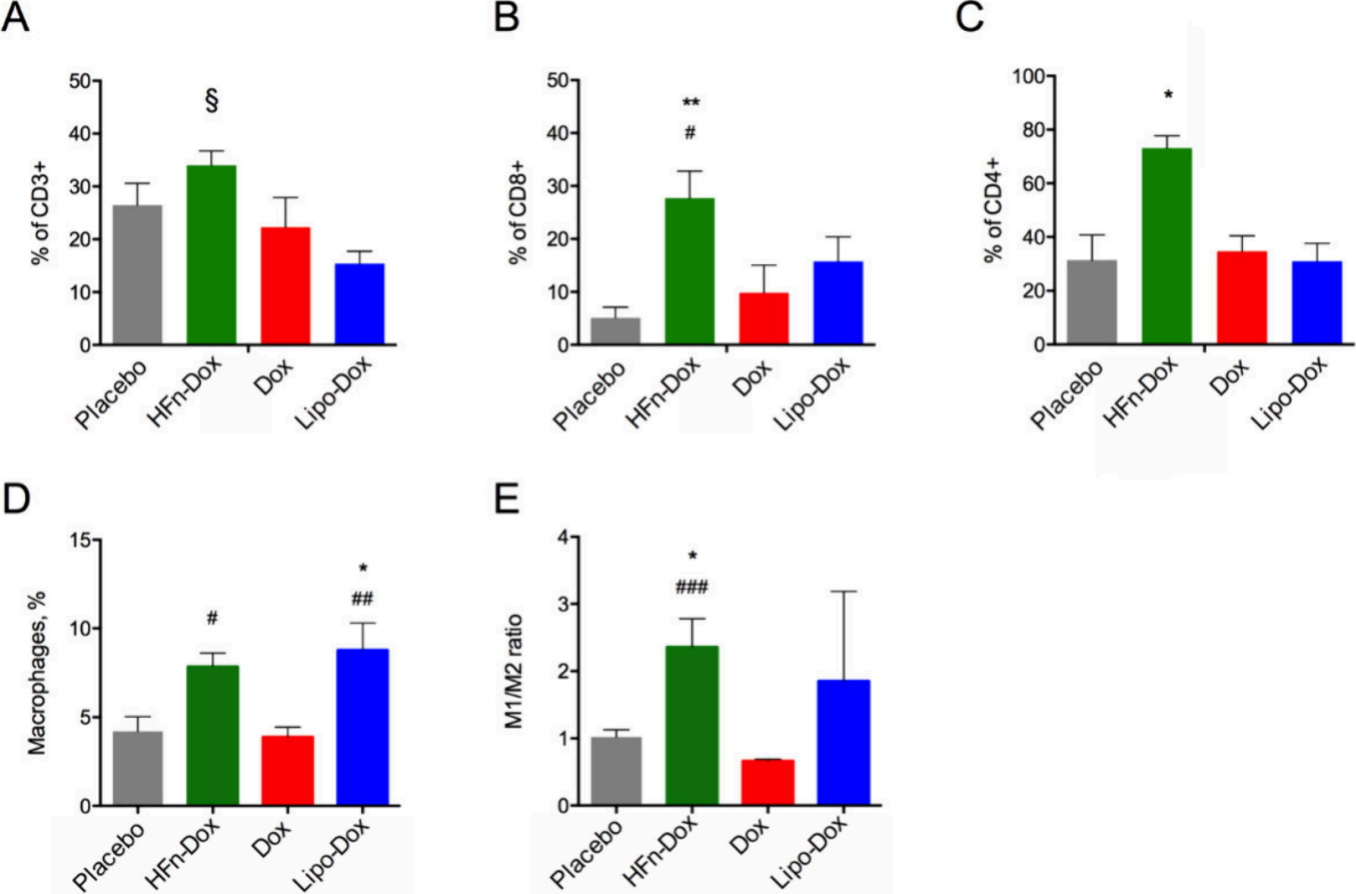
Immunophenotyping of the tumor immune microenvironment in treated mice. The frequency of CD3+ in CD45+ cells (A), CD4+ effectors (B), CD8+ cells (C), total macrophages, (D), M1/M2 ratio (E) were measured as percentage of live cells in tumor homogenates at the end of treatment. Total macrophages were identified as CD11b+ Ly6G– F4/80+ cells; M1 macrophages were identified as CD45+ F4/80+ CD80+ CD86+ INOS+; M2 macrophages were CD45+ F4/80+ CD206+ ARG1+. Data are means ± SE (*n* = 6), § *p* < 0.05, HFn-Dox vs Lipo-Dox, *p < 0.05, **p < 0.01 vs placebo; #*p* < 0.05, ##*p* < 0.01, ###*p* < 0.001 vs Dox by one-way Anova.

### 3.3 HFn-Dox-Mediated Immunomodulation

To further study the immune alterations observed in vivo, we incubated the different Dox formulations with primary T cells and monocytes extracted from the spleen and bone marrow of healthy BALB/c mice. In this way, we could investigate whether the immunomodulation is regulated by different interactions between the immune cells and the drugs. Results showed that all three compounds altered the viability of splenic T cell. In particular, free Dox killed more T cells as compared to HFn-Dox and Lipo-Dox, resulting in less than 50% of residual alive cells after treatment (Figure 6A). Of note, Dox treatment significantly reduced the CD4+/CD8+ ratio (Figure 6B) indicating a preferential effect of Dox toward CD8+ highly proliferating cells. By contrast, HFn-Dox and Lipo-Dox significantly preserved T cells. We also observed that free Dox triggered an increase in PD1 expression on both CD4+ and CD8+ T cells, indicating T cell exhaustion and loss of effector functions upon treatment. By contrast, HFn-Dox and Lipo-Dox limited PD1 induction on T cells (Figure 6C, D), thus, suggesting potential preservation of their functionality.

**Figure 6:**
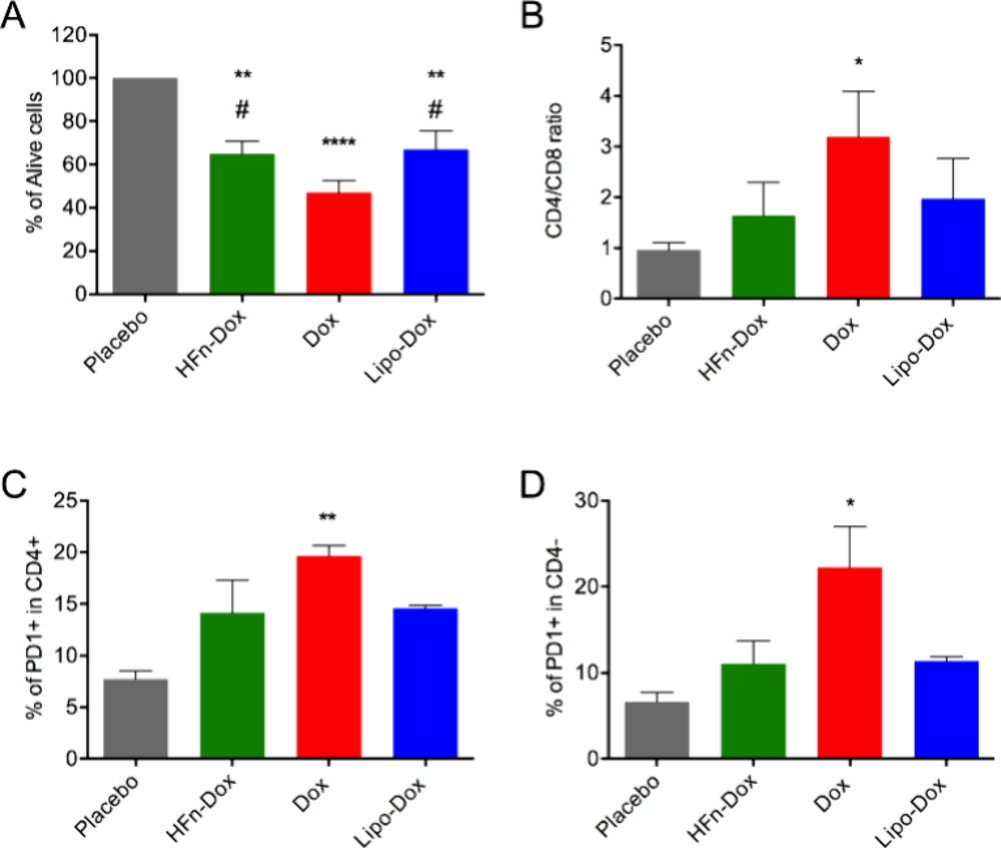
In vitro treatment of splenic T cells. A) Percentage of alive splenocytes upon incubation with HFn-Dox, free Dox and Lipo-Dox equally dosed; B) CD4+/CD8+ ratio (* *p* = 0.026, Mann–Whitney test Dox vs placebo); C, D) mean percentage of PD1+ in CD4+ or in CD4- cells. Data are means ± SE (*n* = 6); *p < 0.05, **p < 0.01, **** *p* < 0,0001 vs placebo; # *p* < 0.05 vs Dox (2-way Anova).

After studying immunomodulatory effects on T cells, we investigated whether there was an effect on dendritic cells and macrophages differentiated in vitro from murine bone marrow-derived monocytes. Results showed that treatment with free Dox, but not HFn-Dox and Lipo-Dox, significantly reduced viable macrophages and dendritic cells after in vitro differentiation (Figure 7A, E), likely due to a cytotoxic effect of the drug toward bone marrow cells. Among the different compounds, HFn-Dox was the only treatment able to promote activation of dendritic cells and macrophages, as assessed by increased expression of CD80+ (Figure 7B, F), CD86+ (Figure 7C, G) and MHCII (Figure 7D, H) on living cells. These data suggest that treatment with HFn-Dox may favor costimulation activity of dendritic cells and macrophages toward T cells, boosting the antigen-specific immune response.

**Figure 7:**
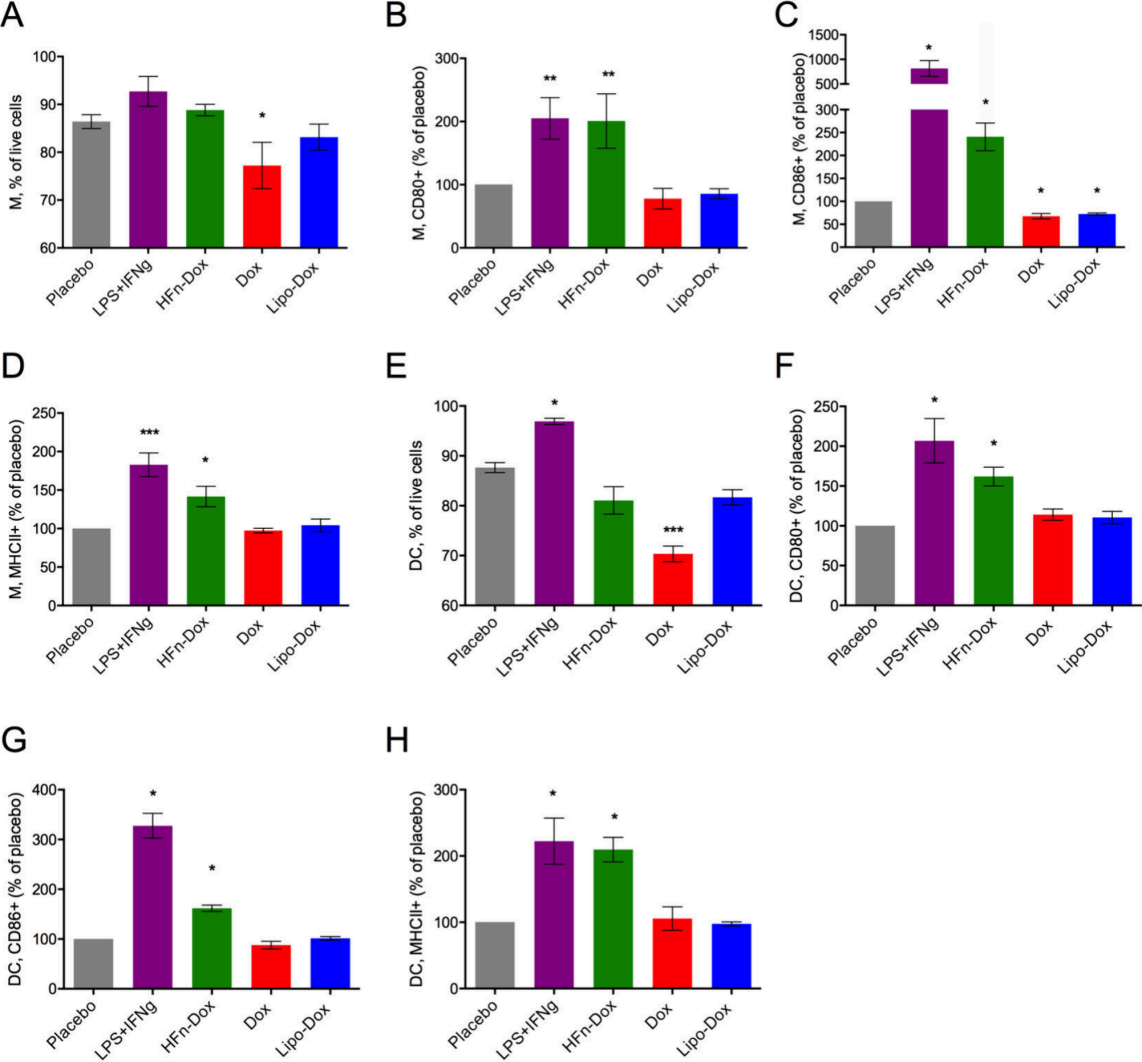
In vitro treatment of bone marrow-derived monocytes differentiated into Dendritic Cells (DC) or Macrophages (M). A) and E) Percentage of live DC or M measured upon treatment with the different compounds. Mean expression of CD80 (B, F), CD86 (C, G) and MHCII (D, H) in live DC or M treated with the different compounds was calculated as percentage of placebo. Data are means ± SE (*n* = 6), *p < 0.05, **p < 0.01, ***p < 0.001 vs placebo by one-way Anova.

## 4 Discussion

TNBC poses a clinical challenge due to its aggressive nature, lack of targeted therapies, and high recurrence rates.^1,31,32^ Previous literature data documented the efficacy and advantages of using H-ferritin-nanocaged doxorubicin (HFn-Dox) in the metronomic treatment of TNBC, also showing reduced cardiotoxicity as compared to free Dox and Lipo-Dox.^14,18^ Here we tested its use as standard-dose chemotherapy in both a syngeneic 4T1 model of TNBC and, for the first time, in a PDX. This is particularly relevant as the treatment of aggressive tumors like TNBC often relies on high-dose chemotherapy, where the adverse effects of free Dox frequently limit its use due to severe toxicity.^33,34^

Results demonstrated superior therapeutic performance of HFn-Dox over free Dox after iv administration in preclinical animal models of TNBC, as demonstrated by reduced tumor cell growth and metastasis formation. Significant advantages of HFn-Dox included increased tolerability to the treatment and protection of the cardiac mitochondria, further confirming a favorable safety profile. Although the Lipo-Dox formulation also shows efficacy in reducing tumor volume, it carries cardiac mitochondria damage, which is a prominent and dangerous side effect associated with patients under treatment.^35,36^

HFn-Dox show the great advantage of being naturally targeted to the tumor cells through specific binding with TfR1.^12,37^ As shown by the extensive data available in the Human Protein Atlas and the literature,^38^ TfR1 is generally expressed at low levels throughout the body, with high expression restricted to the lung, placenta, and bone marrow. Most cancer tissues exhibit moderate to high TfR1 positivity. Indeed, several recent studies support its role as a cancer biomarker, prognostic factor, and therapeutic target.^39–41^ Furthermore, TfR1 is widely expressed in various cancers, including breast cancer, as demonstrated by TfR1 immunohistochemistry of clinical samples from the Human Protein Atlas.^38^ To date, biodistribution studies conducted by our group^14^ and others have shown tumor-specific accumulation of HFn in tumor-bearing mice, with off-target distribution limited to the liver and kidneys. This suggests that TfR1 expression in tumors may be higher than that observed in the lungs and bone marrow of healthy individuals. Moreover, in vitro studies have demonstrated that HFn can specifically bind to and be internalized by a panel of human and murine cancer cells,^12,14,16–18^ while its interaction with healthy cells is negligible. By this mechanism, HFn-Dox are able to avoid toxicity in off target organs like the heart and bone marrow, further allowing scaling up of the administered dosage while keeping safety. In contrast, free Dox and Lipo-Dox rely mainly on passive accumulation via the enhanced permeability and retention (EPR) effect and lack specific targeting capabilities. As a consequence, they may still extravasate into nontumoral tissues with leaky vasculature or be taken up nonspecifically, potentially leading to higher off-target exposure and toxicity.^42^

It has to be noted that the efficacy of HFn-Dox differs between those of the syngeneic 4T1 model and the PDX system. Such variability is indeed expected, as different preclinical models may exhibit heterogeneous responses to the same therapeutic formulation due to tumor–intrinsic factors and host–microenvironment interactions. A first possible explanation relates to the intrinsic drug sensitivity of the tumor cells. It is well established that 4T1 cells are particularly prone to develop resistance to doxorubicin, partly due to their ability to upregulate drug efflux pumps such as P-glycoprotein (MDR1).^14,43^ In contrast, the IC50 values of the PDX model employed in this study are not available, but PDXs are generally considered to retain the chemosensitivity profile of the patient tumor.^44^ This difference may contribute to the relatively higher efficacy observed in the PDX setting. Second, the tumor microenvironment may play an important role. Ferritin nanoparticles, including HFn-Dox, have been shown not only to enter tumor cells via TfR1-mediated uptake but also to interact with stromal components, such as cancer-associated fibroblasts (CAFs), which can influence drug delivery and therapeutic outcome.^18^ The composition and density of the stromal compartment differ substantially between 4T1 tumors and human-derived xenografts, possibly modulating drug efficacy in distinct ways. In PDX, we observed that HFn-Dox induced an increase in tumoral fibrovascular stroma and inflammatory cell infiltration (Figure 2C,D), while this effect was not observed in 4T1 tumors (data not shown), probably due to a different composition and different tumor-TME interaction between the models. This may have influenced the overall response of the 4T1 tumor or PDX to the tested formulations. Another critical factor concerns the immune system. In the immunocompetent 4T1 model, HFn-Dox showed reduced antitumor efficacy compared to Lipo-Dox. This may be linked to pharmacokinetic differences. Liposomal formulations are known to evade rapid clearance by the reticuloendothelial system (RES), thereby prolonging circulation time and enhancing tumor accumulation via the enhanced permeability and retention (EPR) effect.^42^ Conversely, HFn-Dox, being a protein-based nanocarrier, may be more susceptible to clearance in immunocompetent hosts, leading to lower bioavailability and reduced efficacy.^45^ Such a phenomenon could explain the discrepancy in therapeutic outcomes between the two models, although a dedicated biodistribution study in the PDX model was not performed in this work.

Interestingly, our data suggest that HFn-Dox can modulate the tumor immune microenvironment, potentially creating a more responsive setting. This is particularly relevant in recent years, where the growing prominence of immunotherapy has underscored the critical need to understand and mitigate the immunosuppressive effects triggered by high-dose chemotherapy. Immunotherapy, which relies on the activation and engagement of the immune system to fight cancer, can be significantly hindered by the immunological reset caused by chemotherapy.^46–48^

Findings from the present study indicate that HFn-Dox treatment promotes intratumoral T cells and M1-like macrophage polarization. Moreover, HFn-Dox also preserved T cell functionality as demonstrated by reduced expression of PD-1 in vitro. This is a significant concern in cancer therapy because through PD1-PDL1 interaction the tumor can limit the effectiveness of the immune system in recognizing and destroying cancer cells and contribute to shape an immune suppressive microenvironment.^49–52^ We reasoned that using HFn-Dox may help preserve T cell function by reducing aspecific distribution of the drug in the immune cells and possibly avoiding T cells exhaustion. Beyond T lymphocytes, results from our study suggest that HFn-Dox may push the balance between M1 and M2 polarized macrophages toward the M1 phenotype. Tumor-associated myeloid cells constitute a series of plastic and heterogeneous cell populations that exhibit different phenotypes and functions in response to various microenvironmental signals.^53–56^ In general, M1 macrophages promote inflammatory responses against invading pathogens and tumor cells, whereas M2 macrophages tend to exhibit an immunosuppressive phenotype, favoring tissue repair and tumor progression.^57–59^

While the precise mechanisms underlying HFn-Dox immunostimulatory effect remain to be fully elucidated, previous studies have shown that HFn-Dox promotes dendritic cell maturation and enhances expression of costimulatory molecules in a hepatocellular carcinoma model.^60^ This is consistent with our current in vitro observations, where HFn-Dox treatment increased costimulatory molecule expression on dendritic cells. Importantly, we also observed that HFn-Dox preserves T cell viability and function, likely due to reduced uptake of the drug in these cells, confirming findings from our previous work.^20^ These observations suggest that HFn-Dox may indirectly favor M1 macrophage polarization via activation of antigen-presenting cells while sparing T cells from cytotoxic effects, although further studies are needed to fully confirm this mechanism.

To our knowledge, this is the first study of HFn-Dox in a PDX model, which provides critical confirmation and insights into the therapeutic potential of HFn-Dox in a clinically relevant setting. Key findings include increased efficacy in inducing tumor regression in a primary human tumor naïve to prior therapies, enhanced tumor-specific DNA damage, and the absence of documented cardiotoxic, hepatotoxic, and nephrotoxic effects even when the administered dose of the nanodrug was doubled. While the PDX model did not allow analysis of the adaptive immune system activation, a marked peritumoral inflammatory infiltrate was observed upon HFn-Dox treatment, suggesting an alteration of the tumor microenvironment. This immune-modulatory effect was distinct and not seen with the other formulations, highlighting the unique properties of HFn-Dox delivery.

## 5 Conclusions

The results shown in this study highlight the valuable potential of standard-dosed HFn-Dox as a promising approach for the treatment of TNBC. HFn-Dox treatment is associated with reduced side effects mainly on cardiac and immune cells and allows an increase in the drug dosage as compared to conventional chemotherapy formulations. Further translational studies are warranted to assess the clinical feasibility and potential synergistic effects of combining HFn-Dox with other therapeutic modalities for the benefit of patients affected by TNBC.

## Supplementary Material



## Data Availability

All data generated or analyzed during this study are included in this published article and its Supporting Information files. The raw data sets are available from the corresponding author on request.

## Notes

Figure S1_ Gating strategy for flow cytometry analysis; Figure S2_Additional data on HFn-Dox DLS analysis; Figure S3_In vitro cytotoxicity evaluation of HFn-Dox; Figure S4_Analysis of lung metastases in PDX; Figure S5_Quantification of γH2AX+ cells in PDX tumors; Figure S6_Relative body weight of PDX over treatment; Figure S7_Hematoxylin-eosin images of liver and kidneys from the PDX study; Figure S8_4T1 tumor weight ex vivo; Figure S9_Tolerability of HFn-Dox in 4T1-bearing mice; Figure S10_Analysis of splenic T cells from treated 4T1-bearing mice (PDF)

## Notes

Animals were handled in accordance with an experimental study approved by the Italian Ministry of Health (aut. N. 110/2018-PR). The procedures followed were in accordance with the ethical standards of the WMA Statement on Animal Use in Biomedical Research.
