# Supplementary material for: Antitumor Efficacy and Immunomodulation of H‑Ferritin Nanocaged Doxorubicin for Triple Negative Breast Cancer
Source: ACS Appl Nano Mater. 2025 Nov 3;8(45):21724–37. doi: 10.1021/acsanm.5c03120 (PMC12834160; doi:10.1021/acsanm.5c03120)

Supporting Information

Antitumor Efficacy and Immunomodulation of H-Ferritin Nanocaged Doxorubicin for Triple Negative Breast Cancer

Marta Truffi[1], Leopoldo Sitia[2], Serena Mazzucchelli[2], Marta Sevieri[2], Arianna Bonizzi[2], Francesco Mainini[2], Raffaele Allevi[2], Simone Canesi[3,4], Camilla Recordati[3,4], Angelica Stranieri[4], Saverio Paltrinieri[4], Carlo Francesco Morasso[1], Francesca Baldelli Bombelli[5], Fabio Corsi[1,2*]

[1] Istituti Clinici Scientifici Maugeri IRCCS, via Maugeri 4, 27100 Pavia, Italy

[2] Dipartimento di Scienze Biomediche e Cliniche, Università di Milano, via G.B. Grassi 74, 20157 Milano, Italy

[3] Mouse and Animal Pathology Laboratory, Fondazione Unimi, viale Ortles 22/4, 20139 Milano, Italy

[4] Dipartimento di Medicina Veterinaria e Scienze Animali, Università di Milano, via dell'Università 6, 26900 Lodi, Italy

[5] SupraBioNanoLab, Department of Chemistry, Materials, and Chemical Engineering "Giulio Natta", Politecnico di Milano, Milano, 20131, Italy

* Corresponding author: Fabio Corsi, Associate Professor of General Surgery, Dipartimento di Scienze Biomediche e Cliniche, Università di Milano, via G.B. Grassi 74, 20157 Milano, Italy, phone: +39 0250319858, email: fabio.corsi@unimi.it; Head of Breast Unit, Surgery Department, Istituti Clinici Scientifici Maugeri IRCCS, Via Maugeri 4, 27100 Pavia, Italy, phone: +39 0382592272, email: fabio.corsi@icsmaugeri.it

Figure S1: Gating strategy for flow cytometry analysis. Representative flow cytometry plots showing the sequential gating strategy used to identify viable immune cell subpopulations from dissociated 4T1 tumors. Gating was performed on singlets and live cells, followed by sequential selection of CD45+ leukocytes and further characterization using antibody panels specific for myeloid cells (panel 1), lymphoid subsets (panel 2), T cell activation/exhaustion markers (panel 3), and macrophage polarization markers (panel 4).

Panel 1

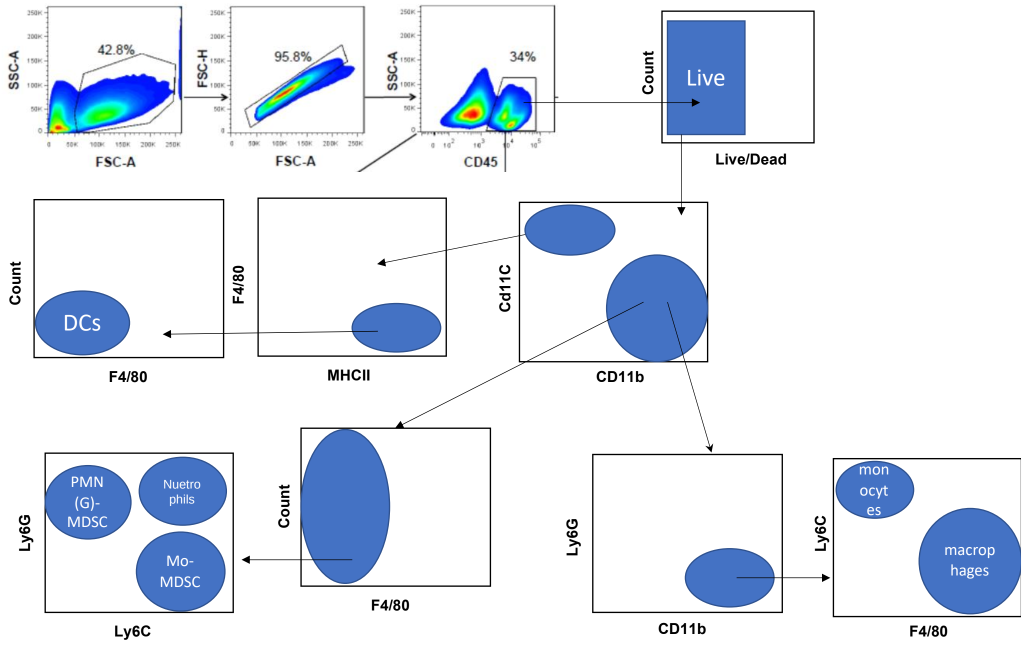

Panel 2

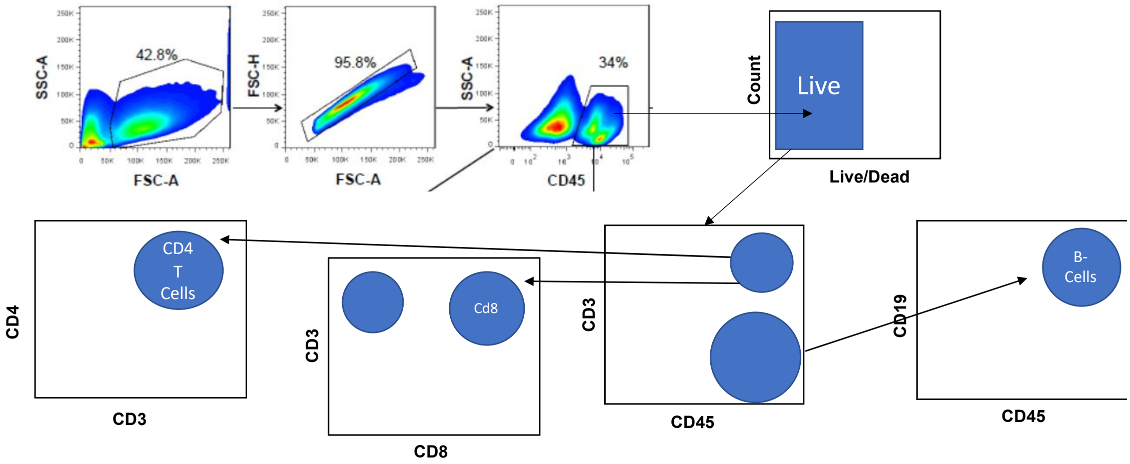

Panel 3

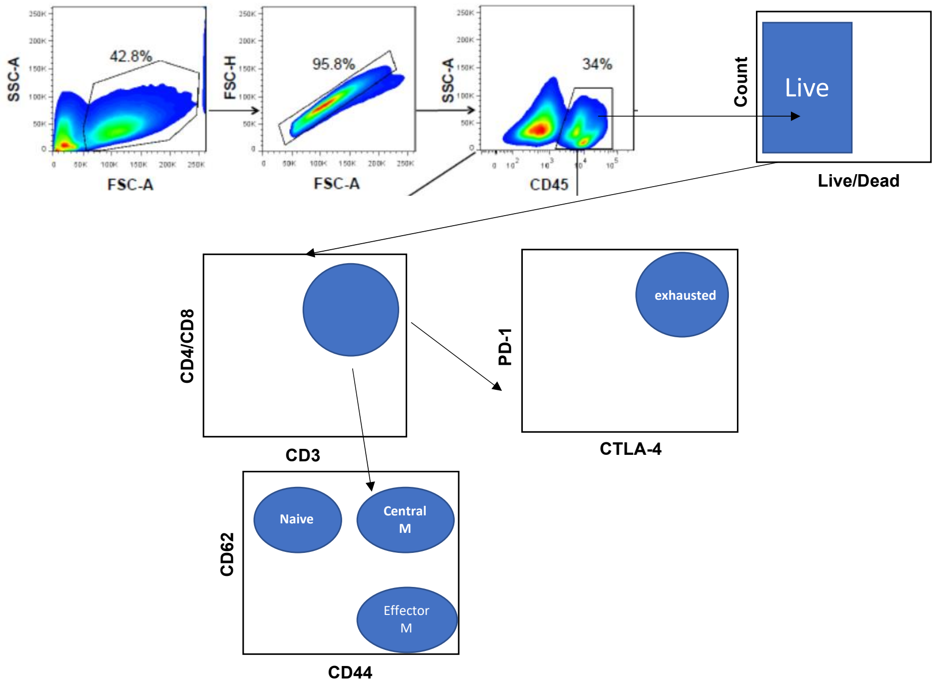

Panel 4

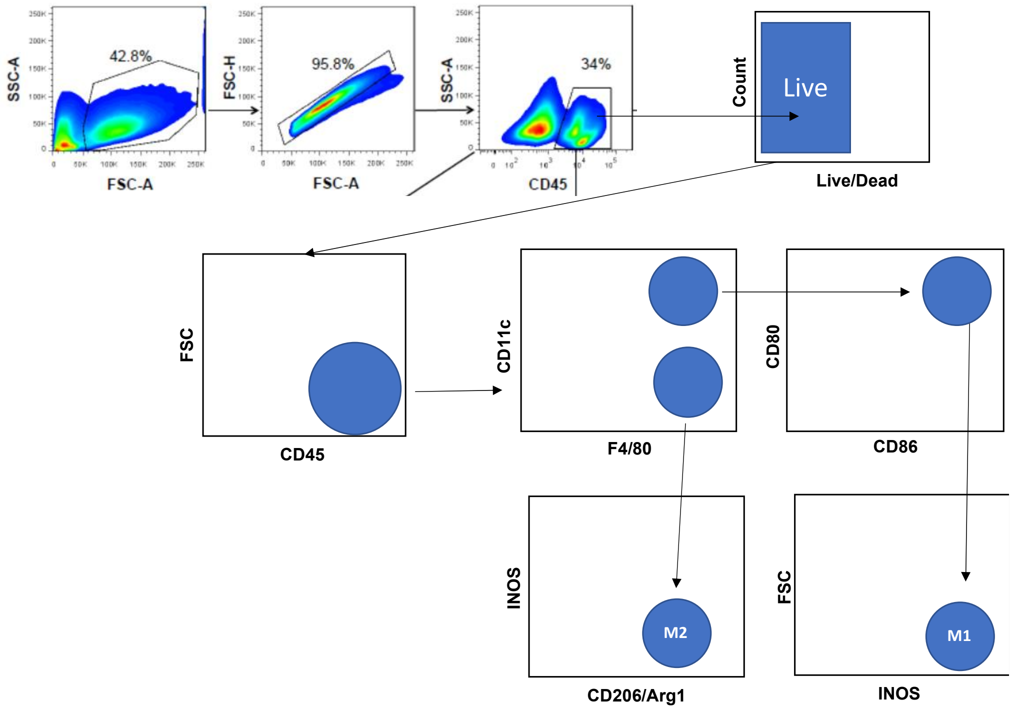

Figure S2: Normalized intensity spectrum of HFn (A) and HFn-Dox (B) radius distribution obtained by DLS analysis. (C) Autocorrelation function from HFn and HFn-Dox samples.

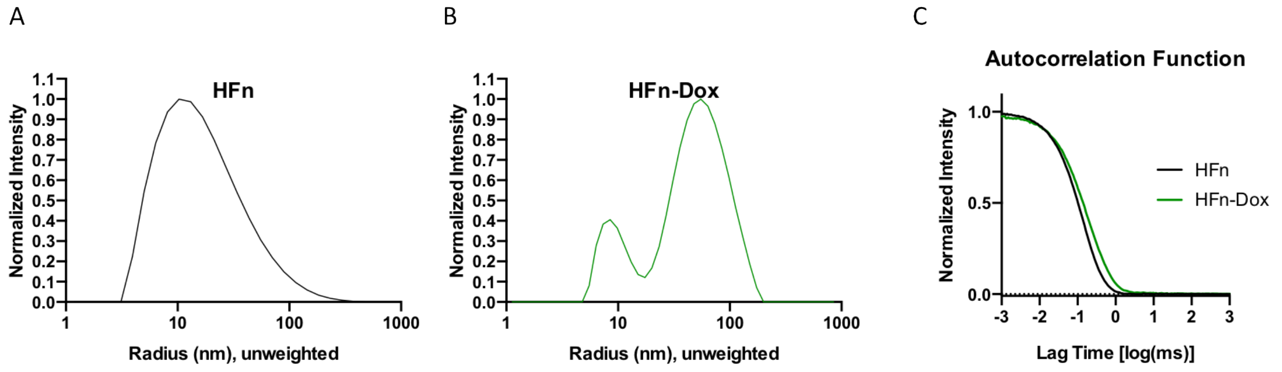

Figure S3: Cytotoxicity evaluation of 4T1-Luc2 cells treated with HFn-Dox and Dox at increasing concentrations (0.01, 0.1, 1, 10 μg/mL) for 24 and 48 h (MTS assay).

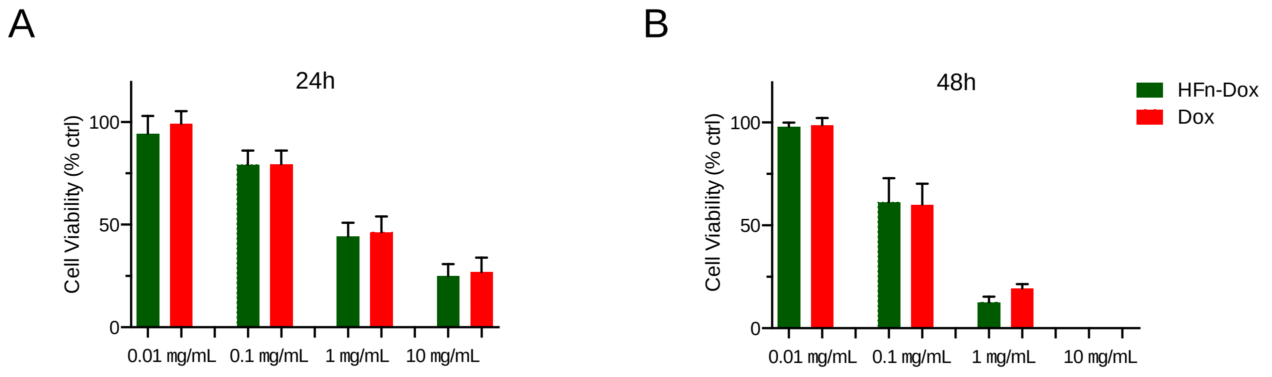

Figure S4: Lung metastases in PDX mice. A) Histology of the lung of a placebo mouse (H&E staining). B) Percentage of mice with MHCI+ cells detected in the lungs (n = 4).

A

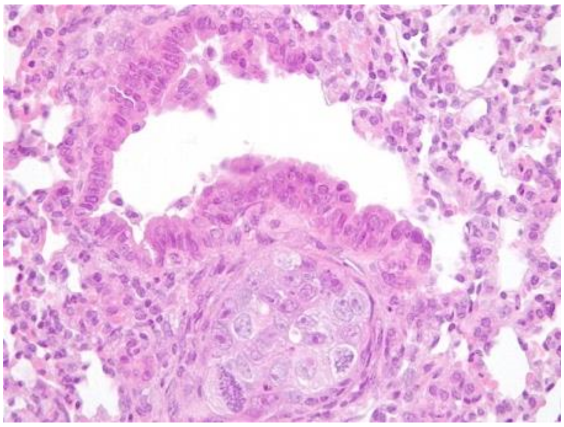

B

**Group**	**n**	**Metastatic MHCI+ cells (% affected mice)**
Placebo	4	100%
HFn-Dox 2.5	4	0%
HFn-Dox 5	4	0%
Dox 2.5	4	25%
Lipo-Dox 2.5	4	0%

Figure S5: Quantification of yH2AX+ cells as percentage of total cells in PDX tumors.

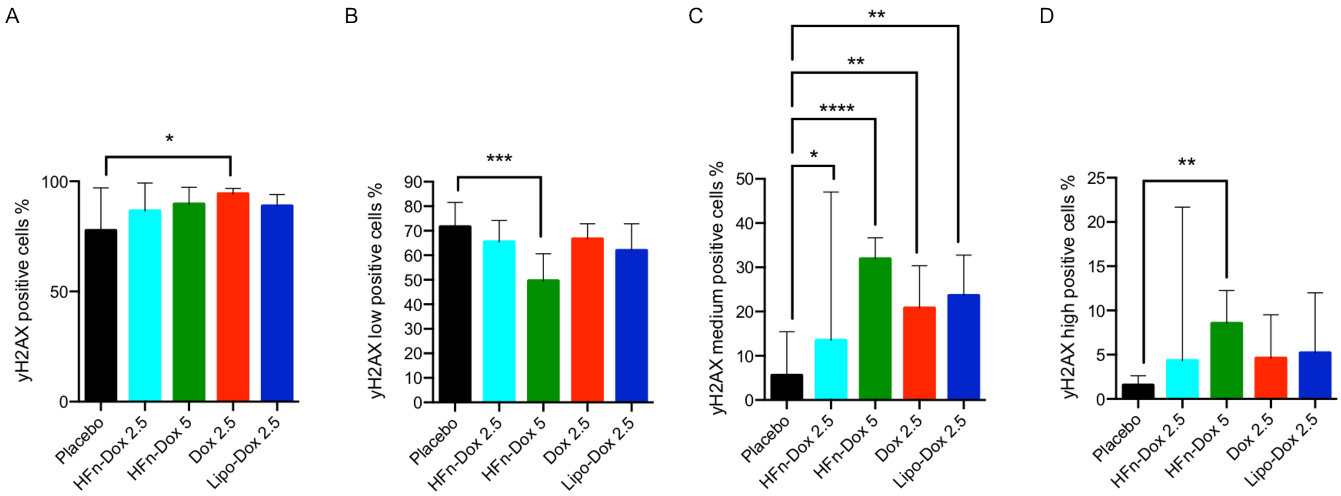

Figure S6: Relative body weight of PDX mice evaluated longitudinally from D0 to D21.

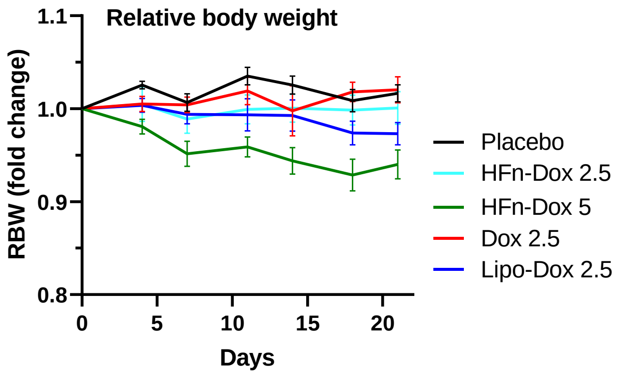

Figure S7: PDX model. Representative images of liver and kidney, no treatment-related effects were observed. H&E, 40x objective. Scale bar = 50 µm.

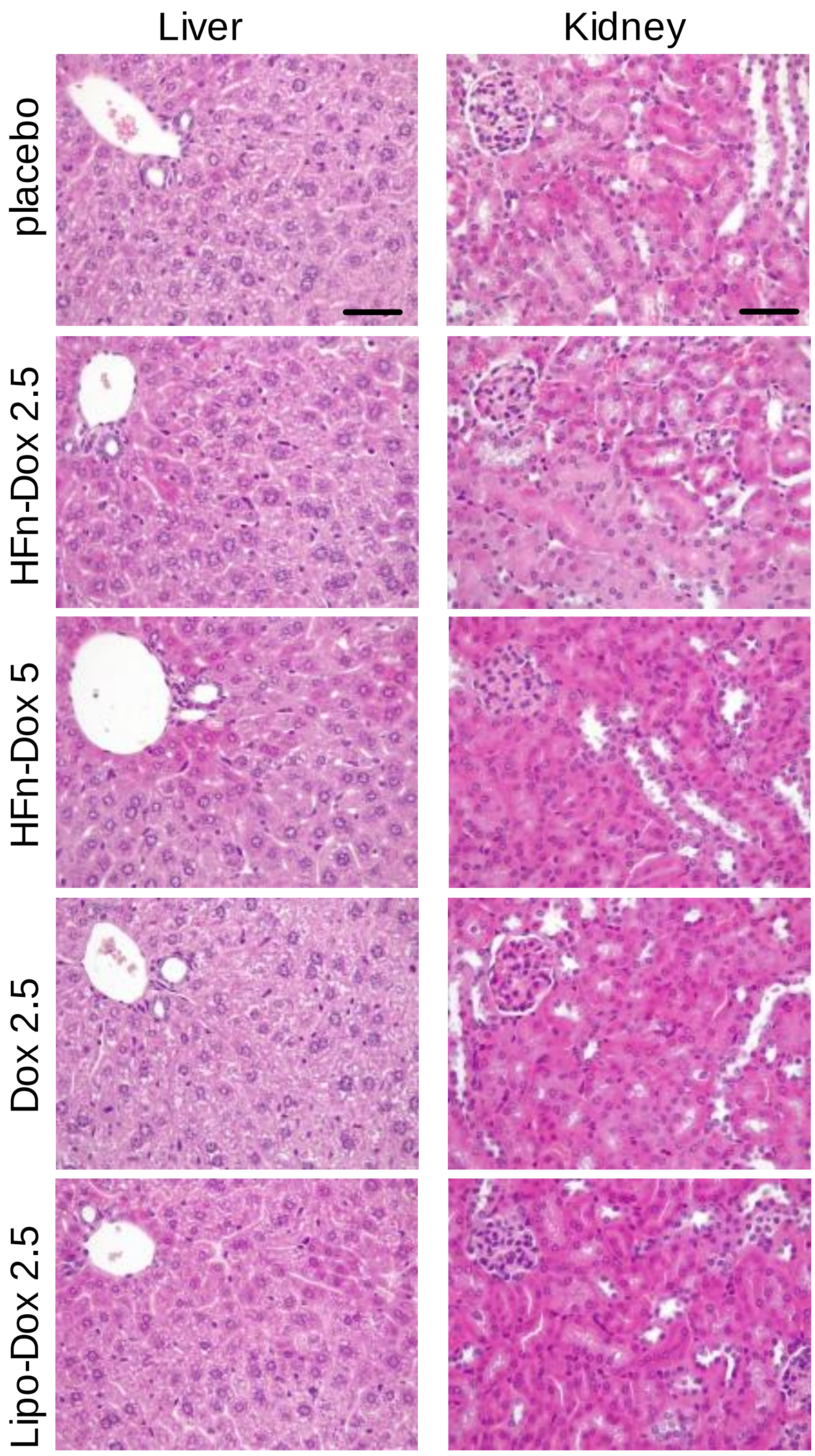

Figure S8: 4T1 tumor weight ex vivo at the end of treatment. Plot reported individual values ± SE. Statistical significance ***p<0.001, ****p<0.0001 vs. placebo; §§p<0.01 vs. Lipo-Dox by one-way Anova.

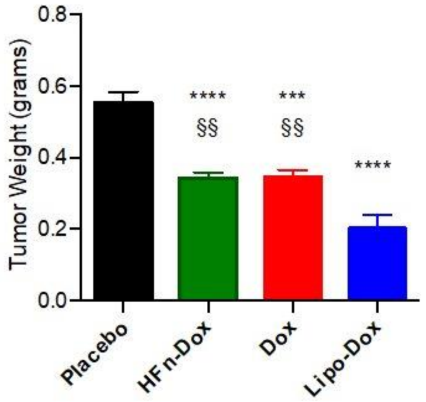

Figure S9: Tolerability of HFn-Dox treatment in 4T1-bearing mice. A) Animal body weight was assessed at day 4, 7, 11, 14, 18 post tumor implant and changes during treatment were reported as percentage of the initial weight before treatment; data are means ± SE (n = 10). B) Hemoglobin concentration and C) white blood cells were measured by blood counts at the end of treatment. Data are means ± SE (n = 6), **p<0.01, ****p<0.0001 vs. placebo; #p<0.05, ##p<0.01 vs. Dox by one-way Anova.

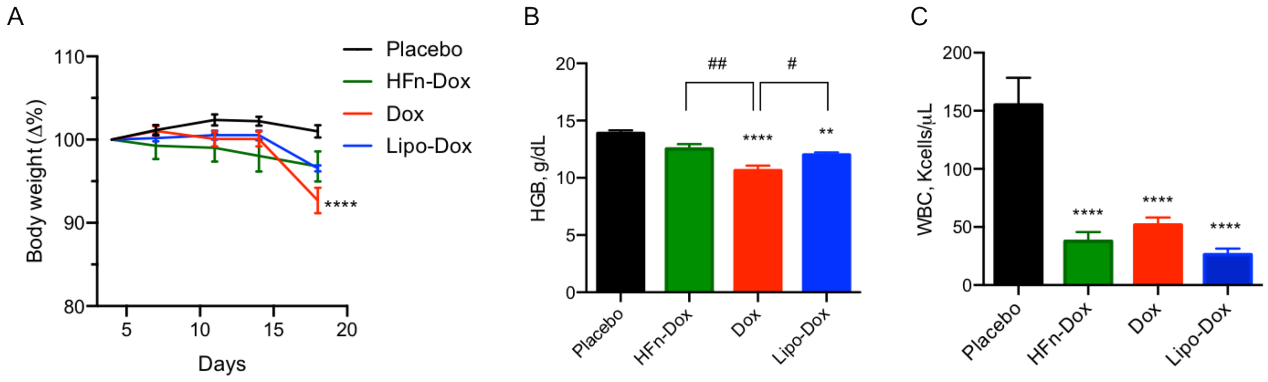

Figure S10: Analysis of splenic T cells from 4T1-bearing mice treated with HFn-Dox, Dox or Lipo-Dox.

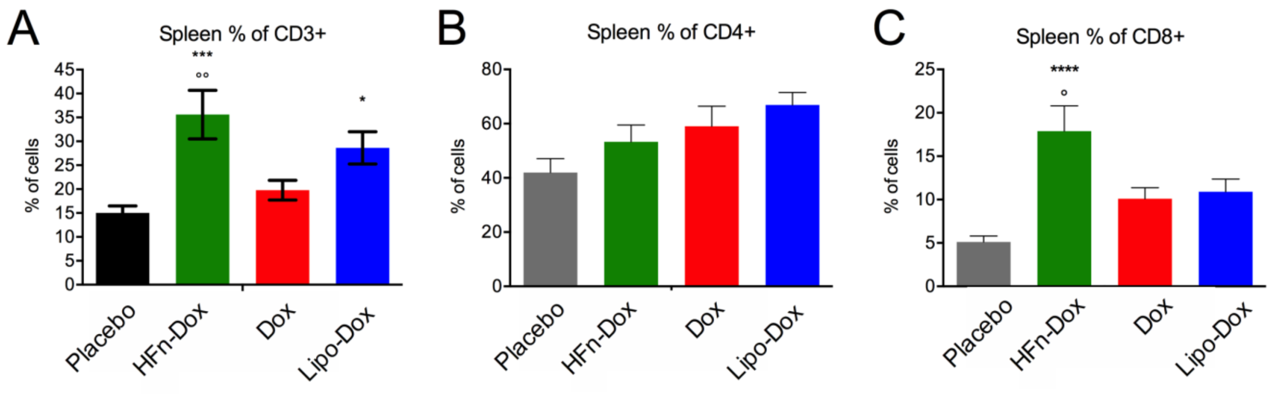

Supplement: Supplementary file 1 [file an5c03120_si_001.pdf]
